# “Double-hit” precipitates fulminant cardiac dysfunction in a child with homozygous CAP2 variant: a case report

**DOI:** 10.3389/fcvm.2026.1846316

**Published:** 2026-06-09

**Authors:** Zhenhui Pan, Jiaojiao Wan, Kaiyu Zhou, Min Tan, Yifei Li

**Affiliations:** 1 Key Laboratory of Birth Defects and Related Diseases of Women and Children of MOE, Department of Pediatrics, West China Second University Hospital, Sichuan University, Chengdu, Sichuan, China; 2 Key Laboratory of Birth Defects and Related Diseases of Women and Children of MOE, Department of Pediatric Cardiovascular Nursing, West China School of Nursing, West China Second University Hospital, Sichuan University, Chengdu, Sichuan, China

**Keywords:** CAP2, dilated cardiomyopathy, distinguish diagnosis, double hits, exome sequencing

## Abstract

**Background:**

Clinical evidence increasingly supports that in children harboring underlying dilated cardiomyopathy (DCM) associated genetic variants, respiratory viral infection frequently serves as the unmasking trigger, precipitating fulminant cardiac dysfunction. Herein, we report a case of DCM with a homozygous loss-of-function *CAP2* variant which was firstly identified by virus infection triggered fulminant cardiac dysfunction. This case reinforces the link between cytoskeletal dysfunction and cardiac electromechanical failure, while also expanding the adverse impacts of virus infection in DCM long-term management.

**Case presentation:**

The proband was a 5-year-old boy presented fulminant cardiac dysfunction. Respiratory pathogen panel was positive for rhinovirus. Exome sequencing (ES) identified a novel homozygous variant in *CAP2* c.551G > A (p.W184*; NM_006366.3). The nonsense mutation resides within the N-terminal helical folded domain, resulting in complete truncation of the central and C-terminal regions. Abrogation of WH2 domain is predicted to completely abolish CAP2 function, impairing sarcomeric actin recycling and cytoskeletal integrity. Sustained intensive therapies could reverse ventricular remodeling conferred clinically significant functional benefit in this patient. Rather, the clinical trajectory of this patient is best interpreted through the lens of a “double-hit” pathogenic mechanism.

**Conclusion:**

To our knowledge, this is the first report of the novel homozygous CAP2 c.551G > A (p.W184; NM_006366.3) variant associated with pediatric DCM. And the early genetic screening should be prioritized in children presenting with unexplained cardiac malformation, growth retardation, or a family history of consanguinity. Moreover, infection prevention and prompt intervention, including adherence to recommended vaccination schedules, are paramount in known DCM carriers.

## Introduction

Dilated cardiomyopathy (DCM) is a myocardial disorder characterized by left ventricular or biventricular dilation accompanied by progressive impairment of systolic function. It demonstrates substantial heritability, with variants across more than a large number of genes implicated in its pathogenesis. These genetic alterations disrupt myocardial structural integrity, metabolic homeostasis, or intracellular signaling cascades, ultimately culminating in contractile failure, arrhythmias, and overt heart failure. Among the implicated genetic categories, cytoskeleton-related genes have garnered increasing attention in recent years. The cytoskeleton, comprising microfilaments, microtubules, and intermediate filaments, is indispensable for maintaining cellular morphology, facilitating intracellular transport, and regulating cell division and migration (1). Normal cardiomyocyte function depends not only on the coordinated interplay among these structural components but also on a repertoire of accessory proteins that dynamically modulate cytoskeletal organization (2, 3). Actin, the principal microfilament protein in eukaryotic cells, serves as a fundamental driver of cell motility and muscle contraction. To date, six actin-encoding genes have been identified, and pathogenic variants in these genes underlie a spectrum of congenital disorders, including nemaline myopathy (2, 4), cerebral arteriopathy (5), and both dilated and hypertrophic cardiomyopathy (6–8). Beyond actin itself, a growing body of evidence has highlighted the pathophysiological relevance of proteins that regulate cytoskeletal dynamics, encompassing actin polymerization/depolymerization modulators (CAP2, CFL1, PFN1), actin cross-linking and bundling proteins (FSCN1, ACTN1), and motor proteins (MYH1, MYH9) (3, 9).

DCM, however, is not exclusively of genetic origin. Environmental triggers, including viral infections, cardiotoxic agents, and perinatal insults, can independently or synergistically precipitate disease. A landmark 2023 Lancet study formalized this concept through a “double-hit” model: an underlying genetic susceptibility constitutes the first hit, rendering cardiomyocytes inherently vulnerable; a subsequent non-genetic insult then disrupts compensatory mechanisms and activates inflammatory, oxidative, or fibrotic pathways, ultimately precipitating overt DCM (10). Clinical evidence increasingly supports that in children harboring underlying DCM-associated genetic variants, respiratory viral infection frequently serves as the unmasking trigger, precipitating fulminant cardiac dysfunction as the inaugural clinical manifestation, rather than a gradual symptomatic progression. In this context, the genetic substrate silently compromises myocardial reserve, while viral insult overwhelms residual compensatory capacity, rendering the first clinical presentation catastrophically severe (11–13).

Herein, we report a case of DCM with a homozygous loss-of-function (LOF) *CAP2* variant (c.551G > A; p.W184*), which was firstly identified by virus infection triggered fulminant cardiac dysfunction. This case reinforces the link between cytoskeletal dysfunction and cardiac electromechanical failure, while also expanding the adverse impacts of virus infection in DCM long-term management.

## Case presentation

### Clinical presentation and physical examination

The proband was a 5-year-old boy born to consanguineous parents who presented with a 20-day history of progressive fatigue and chest tightness, followed by a 4-day history of worsening nausea and vomiting. At symptom onset, his functional capacity was consistent with New York Heart Association (NYHA) Class II, characterized by mild exertional intolerance with preserved activity at rest. However, his clinical status deteriorated rapidly and dramatically over the ensuing weeks, culminating in NYHA Class IV functional impairment, with the patient becoming bedridden and exhibiting lethargy, acute abdominal pain, anorexia, tachypnea, and persistent emesis. Prior evaluation at an outside institution had documented cardiomegaly and findings consistent with interstitial pulmonary edema on chest imaging, prompting transfer to our center for further management.

Upon admission, the patient appeared acutely ill and mildly obtunded. Vital signs were notable for a temperature of 36.3 °C, heart rate of 112 beats per minute, blood pressure of 93/49 mmHg, and respiratory rate of 36 breaths per minute, consistent with compensated hemodynamic compromise and respiratory distress. Neurological assessment revealed a Glasgow Coma Scale (GCS) score of 12, indicating mild impairment of consciousness. Cardiac auscultation demonstrated muffled heart sounds with a regular rhythm and no audible murmurs, raising concern for pericardial effusion or severely reduced myocardial contractility. Abdominal examination revealed hepatomegaly with the liver edge palpable 3 cm below the right costal margin, of moderate consistency and without tenderness, consistent with congestive hepatopathy secondary to right-sided cardiac dysfunction. The spleen was not palpable. Peripheral examination demonstrated no lower extremity edema, and bilateral dorsalis pedis pulses were palpable, suggesting preserved, albeit tenuous, peripheral perfusion at the time of presentation. However, this patient demonstrated a normal cardiac function with slightly enlarged left ventricle by physical examination three month in advance.

Of note, the patient exhibited poor nutritional status prior (BMI 13.4) to disease onset, with no history of other specific illnesses. The patient had not received routine vaccinations prior to disease onset. No abnormal skin, ocular, or skeletal muscle manifestations were observed on admission.

### Laboratory and imaging evaluation

**Figure 1:**
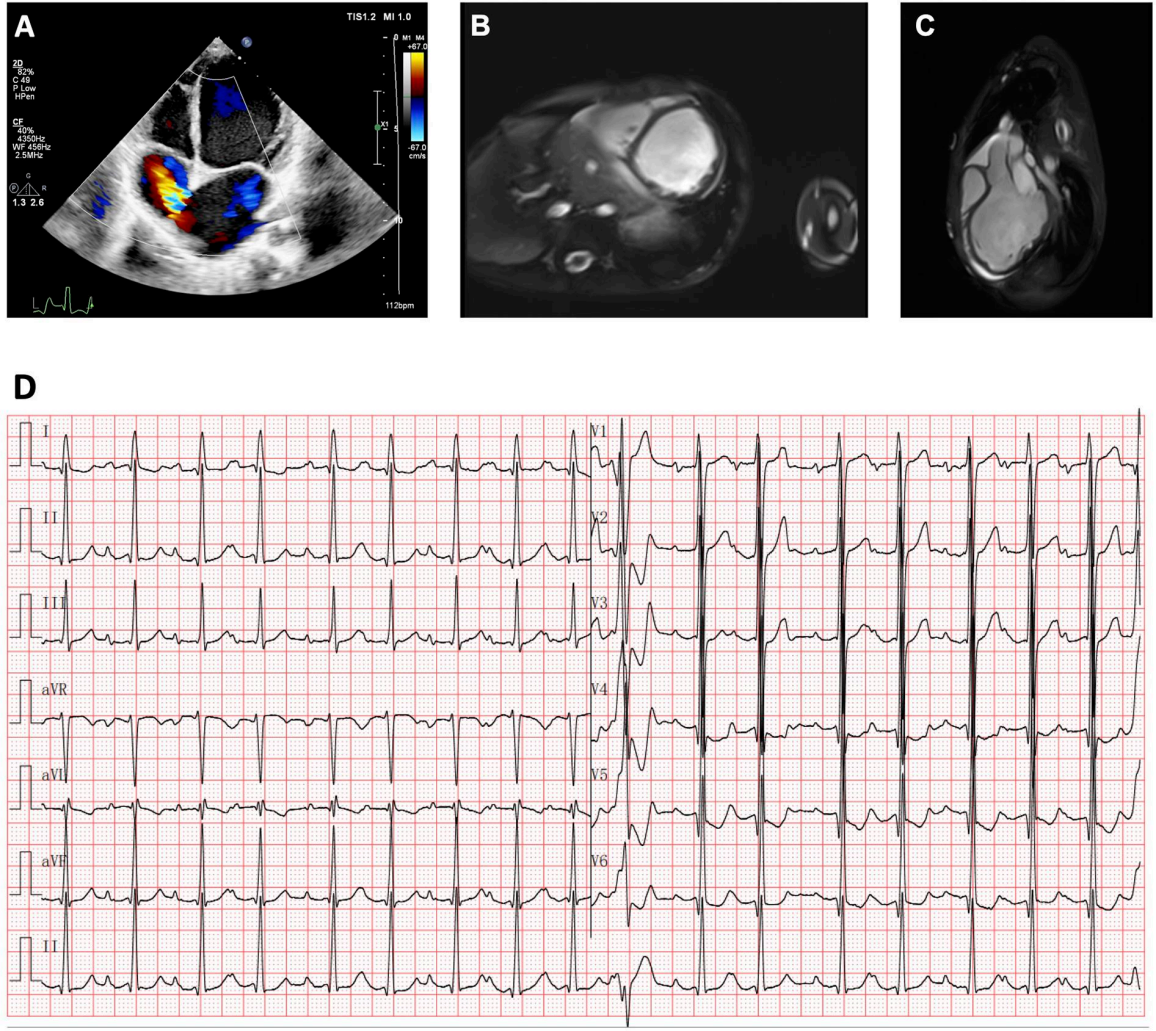
Cardiac imaging and electrocardiogram of the proband. **(A)** Echocardiography showed global cardiac enlargement. Additional findings included an atrial septal defect, mitral valve thickening, and mitral/tricuspid regurgitation. **(B,C)** Enhanced cardiac magnetic resonance imaging showed global cardiac enlargement, predominantly involving the left ventricle, with wall thinning and diminished motion. Additional diffuse foci of delayed enhancement were observed in both ventricles. **(D)** Electrocardiogram showed bilateral atrial abnormality, left ventricular hypertrophy, first-degree atrioventricular block, intraventricular conduction block, and ventricular premature beats.

Laboratory investigations revealed several significant biochemical abnormalities. Serum sodium was markedly reduced at 128 mmol/L (reference: >135 mmol/L), consistent with dilutional hyponatremia in the setting of advanced heart failure and neurohormonal activation. Brain natriuretic peptide (BNP) was profoundly elevated at 4,472.51 pg/mL (reference: <35.00 pg/mL), reflecting severe ventricular wall stress and hemodynamic decompensation. While the detective of cTnI was negative (lower than the reference level of 0.05 ng/mL). Respiratory pathogen panel was positive for rhinovirus. Chest radiography demonstrated bilateral pulmonary infiltrates consistent with pneumonia, increased pulmonary vascular markings indicative of elevated pulmonary venous pressure, and a globally enlarged cardiac silhouette, corroborating clinical findings of cardiopulmonary decompensation. Transthoracic echocardiography revealed global four-chamber enlargement, with a left ventricular end-diastolic dimension of 55 mm, severely reduced wall motion, and markedly impaired systolic function (ejection fraction 32%; fractional shortening 15%). Structural anomalies included an atrial septal defect, mitral valve leaflet thickening, and both mitral and tricuspid regurgitation, these structural anomalies were first diagnosed on admission and had not been recognized prior to the acute cardiac presentation (Figure 1A). Cardiac magnetic resonance imaging (MRI) corroborated echocardiographic findings, confirming global cardiac enlargement with predominant left ventricular involvement, characterized by wall thinning and globally diminished myocardial motion. Notably, diffuse foci of late gadolinium enhancement (LGE) were identified across both ventricles, a pattern strongly indicative of myocardial fibrosis and replacement consistent with dilated cardiomyopathy (Figures 1B,C). Twelve-lead electrocardiography (ECG) demonstrated a constellation of electrophysiological abnormalities, including biatrial enlargement, left ventricular hypertrophy, first-degree atrioventricular block, intraventricular conduction delay, and ventricular premature complexes (Figure 1D), collectively reflecting diffuse electromechanical remodeling. Coronary computed tomographic angiography (CTA) identified a right-dominant coronary system with normal coronary origins and course, and no evidence of luminal stenosis or aneurysmal dilation, effectively excluding ischemic or coronary anomaly-related etiology. Subsequent dedicated imaging confirmed the absence of pericardial effusion and intracardiac thrombus, further delineating the structural and functional disease burden.

### Molecular assessments

Sequencing was performed on the NovaSeq 6000 platform (Illumina, San Diego, CA, USA), and the raw data were processed using FastP to remove adapters and filter low-quality reads. Paired-end reads were aligned to the Ensembl GRCh38/hg38 reference genome using the Burrows–Wheeler Aligner. GATK had been applied to screening SNV and CNV. Variant annotation was performed in accordance with database-sourced minor allele frequencies (MAFs) and practical guidelines on pathogenicity issued by the American College of Medical Genetics. The annotation of MAFs was performed based on the 1000 Genomes, dbSNP, ESP, ExAC, and Chigene in-house MAF database, Provean, Sift, Polypen2_hdiv, and Polypen2_hvar databases using R software (R Foundation for Statistical Computing, Vienna, Austria). However, we did not included the sequencing for mitochondrial DNA as low risk for metabolic disorder had been suspected.

**Figure 2:**
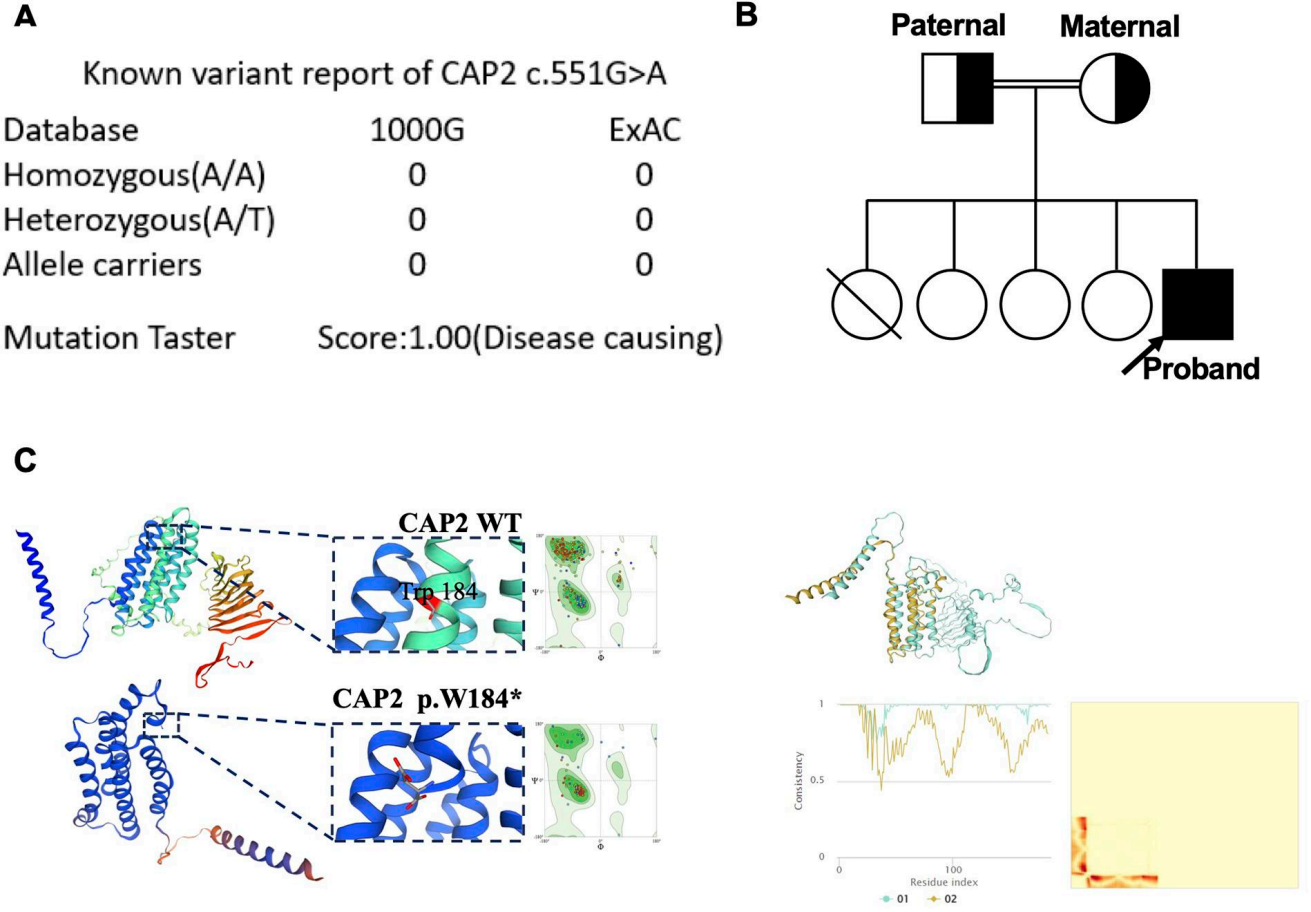
The molecular features of CAP2. **(A)** The variant of CAP2 c.551G > A had never been reported in ExAC and 1000G databases, which was predicted protein damaging by MutationTaster. **(B)** Pedigree of the family. Double horizontal line indicates consanguineous marriage. The proband (arrow) carried a homozygous variant of CAP2 (c.551G > A; p.W184*), while his parents were heterozygous carriers. The proband's older sister (oblique line) died at 3 years old due to unexplained abdominal pain (not genetically tested). No other siblings were available or tested for this variant. **(C)** The protein structure of CAP2 had been built by AlphaFold3 based on the wildtype and mutant sequencing and to compare their structural discrepancies. Then the residues relationship around the mutant site indicating the structure presented due to the variant of CAP2 p.W184*.

The combination of a severe pediatric cardiac phenotype and consanguineous parentage raised a high index of suspicion for an underlying monogenic etiology. Accordingly, trio-based ES was undertaken in the proband and both biological parents. Variant annotation followed ACMG pathogenicity classification guidelines, with MAF filtering performed against the 1000 Genomes Project, ExAC, and an internal Chigene reference database. In silico pathogenicity prediction was conducted using MutationTaster. ES identified a novel homozygous variant in *CAP2* c.551G > A (p.W184*), located within exon 7 of the 13-exon transcript encoding a 477-amino acid protein. This nonsense substitution introduces a premature stop codon at position 184, predicted to cause LOF through either nonsense-mediated mRNA decay or production of a severely truncated non-functional protein. MutationTaster classified this variant as disease-causing with a probability score of 1.00. The variant was entirely absent from the 1000 Genomes, ExAC, and gnomAD popmax databases, with zero homozygous and heterozygous records documented (Figure 2A). Although 511 variants within *CAP2* are catalogued across UniProt, ClinVar, and dbSNP, this specific variant has not been previously reported. This variant is associated with DCM type 2I (OMIM: 620462), and to the best of our knowledge, this case expanded the genotypic spectrum of CAP2-related cardiomyopathy by reporting a novel homozygous c.551G > A variant not previously described.

Segregation analysis confirmed autosomal recessive inheritance, with both parents identified as obligate heterozygous carriers (Figure 2B). Their consanguineous union as first cousins substantially elevated the *a priori* probability of homozygosity for this rare recessive pathogenic allele. As no experimentally resolved full-length CAP2 crystal structure exists, AlphaFold (model: AF-P40123-2-F1-v6) was employed to model the wild-type conformation, with Swiss-Model used for comparative structural analysis between wild-type and mutant proteins (Figure 2C). The nonsense mutation resides within the N-terminal helical folded domain (HFD), resulting in complete truncation of the central and C-terminal regions. This eliminates two functionally critical domains: the WH2 domain, the core G-actin binding interface essential for cytoskeletal dynamics, and the CARP domain, which mediates ADP-to-ATP-G-actin conversion and CAP2 homodimerization. Abrogation of these domains is predicted to completely abolish CAP2 function, impairing sarcomeric actin recycling and cytoskeletal integrity. Additionally, structural modeling revealed an altered β-sheet rotation angle between the first and second α-helices, further compromising residual protein stability. Notably, unlike the classic infantile-onset phenotype reported in CAP2 deficiency (14), our patient presented at 5 years of age with childhood-onset severe dilated cardiomyopathy, for which the above mechanism provides a coherent molecular explanation.

### Final diagnosis and treatment

Based on the integration of clinical, imaging, and genetic findings, the patient received a definitive diagnosis of CAP2-related DCM, with congenital heart defects and acute decompensated heart failure. Genetic confirmation of a homozygous loss-of-function (LOF) variant in *CAP2* confirmed the underlying monogenic etiology, providing a pathophysiological basis for the severe childhood-onset cardiac phenotype observed in this patient. Notably, this variant is associated with dilated cardiomyopathy, consistent with the clinical diagnosis. The patient's mother reported occasional palpitations but had not undergone formal cardiac structural evaluation. No other family members exhibited obvious cardiac symptoms or had received systematic cardiac examinations.

Initial management prioritized hemodynamic stabilization and reversal of acute decompensation. Respiratory compromise was addressed via NIPPV, and broad-spectrum antibiotics were initiated given radiographic pneumonia and rhinovirus co-infection. Guideline-directed medical therapy (GDMT) was systematically instituted, including intensive intravenous inotropic support (milrinone and dobutamine).

And the patient demonstrated a favorable and progressive clinical response to the above therapeutic strategy, with complete resolution of fatigue, chest tightness, and respiratory distress prior to discharge. He was transitioned to an optimized oral heart failure regimen with structured outpatient cardiology follow-up. Notably, serial echocardiographic assessment demonstrated meaningful recovery of left ventricular systolic function, with ejection fraction improving from a nadir of 32% at presentation to 45% at 3 months and 48% at 6 months follow-up respectively. And the classification of NYHA changed into level II both at 3- and 6-months follow-up. And the dosage of medication involved in GDMT kept stable during half a year follow-up. While the level of BNP and repeated cardiac MRI scanning were not provided to the patient. The follow-up results indicated that sustained GDMT and reverse ventricular ejection function conferred clinically significant functional benefit in this patient.

While the observed functional recovery is encouraging, it is important to recognize that the favorable response to GDMT does not negate the underlying genetic vulnerability. Rather, the clinical trajectory of this patient is best interpreted through the lens of a “double-hit” pathogenic mechanism. The first hit represents the homozygous *CAP2* LoF variant, which fundamentally compromises sarcomeric actin dynamics and renders the myocardium inherently susceptible to additional pathological insults. The second hit is represented by the acute rhinovirus infection, which precipitated direct myocardial inflammation and immune-mediated cardiomyocyte injury upon a genetically primed, structurally compromised myocardium. The convergence of these two independent mechanisms is proposed to have precipitated and accelerated the manifestation of decompensated heart failure, underscoring the critical role of environmental triggers in unmasking latent genetic cardiomyopathies. This mechanistic insight further emphasizes the necessity of continued long-term surveillance and proactive therapeutic optimization in patients harboring pathogenic *CAP2* variants, even in the context of apparent clinical stabilization.

## Discussion

*CAP2*, mapped to chromosomal locus 6p22.3, is evolutionarily conserved across mammalian, bacterial, and plant species, yet exhibits notable functional divergence between organisms (Table 1). In mammals, *CAP2* serves as a critical regulator of actin polymerization and depolymerization, participates in cAMP signaling through adenylate cyclase binding, and governs the nuclear translocation of myocardin-related transcription factors (MRTF), thereby modulating downstream serum response factor (SRF) activity. Loss of *CAP2* function results in pathological nuclear accumulation of MRTFB, hyperactivation of SRF, and aberrant overexpression of myosin structural genes, including ACTA1 and Myl9, collectively disrupting sarcomere assembly and driving adverse cardiac remodeling (14–16). These findings are further corroborated by CAP2-knockout murine models, in which SRF hyperactivation was demonstrated to occur selectively within cardiomyocytes rather than cardiac fibroblasts, underscoring the cardiomyocyte-specific pathogenic mechanism of CAP2 deficiency (3, 6, 9, 15).

**Table 1:** Summary of CAP2 domain functional annotations across species.

Species	Core region	Subdomain	Function
Mammals (human, mouse)	N-terminus	CC/OD	Core element for oligomerization; core region for binding to RACK1; mediates CAP2 nuclear localization
		HFD	Key domain for binding to F-actin
	Central region	P1 + P2	Binds SH3-containing proteins and profilin
		WH2	Binds G-actin and promotes ADP-to-ATP conversion
	C-terminus	CARP	Binds G-actin; mediates dimerization; anchors to nuclear matrix
Bacteria (VC, E. cloacae)	N-terminus	E2-like domain	Ubiquitination-like modification
	Central region	–	Maintains protein conformation
	C-terminus	E1-like domain	CD-NTase adenylation; mediates dimerization; activates anti-phage signaling
Saccharomyces cerevisiae	N-terminus	HAP4L domain	Binds HAP complex; recruits to promoters; participates in regulation of iron homeostasis genes
		bZIP domain	Recognizes promoters; regulates transcription of iron homeostasis genes
	C-terminal	–	Synergizes with promoter binding; maintains transcriptional activity

The homozygous variant identified in this case, c.551G > A (p.W184*) in *CAP2* (NM_006366.3, exon 7), introduces a premature termination codon at position 184, directly resulting in a LoF allele through predicted nonsense-mediated mRNA decay or production of a severely truncated non-functional protein (10). Notably, GWAS have previously implicated the 6p21–6p22.3 chromosomal region in susceptibility to sudden cardiac death, hypertension, left ventricular hypertrophy, and coronary artery disease, further contextualizing the pathogenic significance of variants within this locus (15, 17).Clinically, CAP2-related disease is an extremely rare autosomal recessive disorder with only a limited number of cases reported worldwide. CAP2-related disease manifested as a heterogeneous spectrum encompassing DCM (16), structural cardiac anomalies, nemaline myopathy (14), growth retardation, abnormal scarring (9) and microphthalmia (15), frequently accompanied by heart failure, arrhythmias, and neuromuscular complications. Reported structural cardiac abnormalities included left ventricular noncompaction (NCCM), valvular regurgitation, and myocardial fibrosis (14, 16). Most reported cases demonstrated a recognized male predominance in mortality and a high prevalence of parental consanguinity (18).

The clinical course observed in this patient is best interpreted within the framework of the “double-hit” pathogenic model. Under this paradigm, an underlying genetic susceptibility, constituting the first hit, places cardiomyocytes in a chronic compensatory state of structural vulnerability without necessarily producing overt clinical manifestations. A subsequent non-genetic second hit then overwhelms this compensatory reserve, activating inflammatory, oxidative, and fibrotic pathways that precipitate clinical decompensation. In the present case, the homozygous *CAP2* LoF variant represented the first hit, rendering the myocardium inherently susceptible, while acute rhinovirus infection served as the second hit, triggering immune-mediated cardiomyocyte injury and precipitating acute decompensated heart failure. This mechanistic sequence provides a compelling explanation for the patient's prior asymptomatic state followed by rapid clinical deterioration, a pattern consistent with the “silent carrier” phenomenon and the well-documented incomplete penetrance observed among monogenic DCM mutation carriers. Furthermore, the identification of obligate heterozygous carrier status in both consanguineous parents is consistent with autosomal recessive inheritance and reinforces the substantially elevated risk of homozygosity conferred by consanguineous unions, highlighting the critical importance of proactive genetic counseling in at-risk families.

The present case further underscores the pivotal role of respiratory viral infections as precipitating triggers in genetically susceptible individuals, raising important considerations regarding the potential cardioprotective role of vaccination strategies in patients with underlying cardiomyopathy. Accumulating evidence supports the use of influenza vaccination as a cardioprotective intervention in patients with established cardiovascular disease. A landmark randomized controlled trial by Phrommintikul et al. demonstrated that influenza vaccination significantly reduced major adverse cardiovascular events in high-risk cardiac patients, with effects comparable to statin therapy in the short term (19). Subsequent meta-analyses have further corroborated these findings, demonstrating that influenza vaccination is associated with a significant reduction in cardiovascular mortality, hospitalization for heart failure, and acute coronary events among patients with pre-existing cardiac conditions (20). Mechanistically, respiratory viral infections, including rhinovirus and influenza, are recognized to induce systemic inflammatory responses, endothelial dysfunction, and direct myocardial injury, all of which may disproportionately destabilize the already-compromised myocardium in genetically susceptible individuals. In this context, respiratory syncytial virus (RSV) vaccination, now increasingly available for high-risk pediatric and adult populations, may represent an additional preventive strategy worthy of prospective evaluation in patients with hereditary cardiomyopathies (21). Furthermore, COVID-19 vaccination has been associated with reduced risk of myocarditis and cardiovascular complications attributable to SARS-CoV-2 infection itself, reinforcing the broader principle that infection prevention through immunization constitutes a viable and evidence-based strategy for mitigating second-hit events in genetically predisposed cardiac patients (22). Collectively, these data suggest that comprehensive vaccination protocols, encompassing influenza, RSV, and COVID-19 vaccines, should be formally incorporated into the preventive management framework for patients with CAP2-related DCM and other hereditary cardiomyopathies, particularly in the pediatric population where infectious triggers represent a disproportionately significant risk factor for acute decompensation.

To our knowledge, this represents the first reported case of a homozygous CAP2 c.551G > A variant precipitating pediatric DCM, thereby expanding the genotypic and phenotypic spectrum of CAP2-related disease. Several limitations of this study should be acknowledged. First, although rhinovirus infection was identified as the “second hit” in our double-hit model, we did not perform endomyocardial biopsy with viral PCR or serial serum viral load measurements to confirm direct myocardial invasion. Invasive procedures such as biopsy are rarely performed in critically ill pediatric patients due to safety concerns, which represents a common clinical constraint. In addition, fever-induced tachycardia, increased myocardial oxygen demand, and systemic inflammatory responses related to infection may have independently or synergistically contributed to acute cardiac decompensation, beyond direct viral myocardial injury. Three principal clinical implications merit emphasis. First, early genetic screening should be prioritized in children presenting with unexplained cardiac dysfunction, growth retardation, or a family history of consanguinity. Second, infection prevention and prompt intervention, including adherence to recommended vaccination schedules, are paramount in known DCM carriers, given the capacity of infectious triggers to precipitate acute decompensation. Third, despite favorable short-term clinical improvement, long-term surveillance and timely therapeutic adjustment remain essential to mitigate risks of sudden death and end-stage heart failure. Future investigations should delineate the precise role of CAP2 in cardiac development and evaluate the therapeutic potential of targeting the MRTF/SRF pathway, with the ultimate aim of enabling more precise and individualized management strategies for CAP2-related hereditary cardiomyopathy.

## Data Availability

Data supporting the findings of this study are available from the corresponding author upon reasonable request.
